# Molecular Dynamics Simulation of Silicone Oil: Degradation upon Oscillatory Testing

**DOI:** 10.3390/polym18020278

**Published:** 2026-01-20

**Authors:** Pascal Puhlmann, Dirk Zahn

**Affiliations:** Lehrstuhl für Theoretische Chemie/Computer Chemie Centrum, Friedrich-Alexander Universität Erlangen-Nürnberg, Nägelsbachstraße 25, 91052 Erlangen, Germany

**Keywords:** silicone oil, degradation mechanisms, molecular dynamics, cyclic testing

## Abstract

The fate of a selection of linear and cyclic silicone oil formulations in heavy-duty fluid dampers is studied from molecular dynamics simulations. Mimicking cyclic agitation to all-atom simulation models, we elaborate oscillatory compression/decompression runs that feature degradation reactions within only hundreds of loading cycles. This enables the assessment of chain scission, reassembly and cyclization mechanisms from ns-scale molecular dynamics simulations. Using analogous testing scenarios, we compare the degradation reactions of linear and cyclic silicone chains and demonstrate the importance of silicone ring formation. In turn, cyclic silicone moieties show relative persistence in our compression/decompression runs. We conclude that long-term degradation finally leads to a manifold of cyclic silicone molecules, featuring rings of up to tens of monomeric units. The underlying molecules are not inert to Si-O bond cleavage and reformation, but feature reactivity in terms of the fusion of small to large rings and vice versa.

## 1. Introduction

Silicones oils are important alternatives to hydrocarbon-bases lubricants with silicones outperforming alkanes in terms of chemical stability, bio-compatibility and fluidity behaviour under high pressure [1,2,3,4,5]. Much of this is owed to the great stability of the Si-O bonds as compared to C-C bonding in alkanes. This robustness is particularly appealing for heavy-duty application such as lubrication at high temperature or for damping fluids exposed to extreme numbers of testing cycles. In turn, the longevity of silicone oil is limited by only a comparably small range of possible degradation reactions [6,7,8,9,10,11]. In-depth understanding of the underlying mechanisms thus offers the prospect of predicting and ideally controlling the performance of silicone oil formulations [12,13].

**Figure 1:**
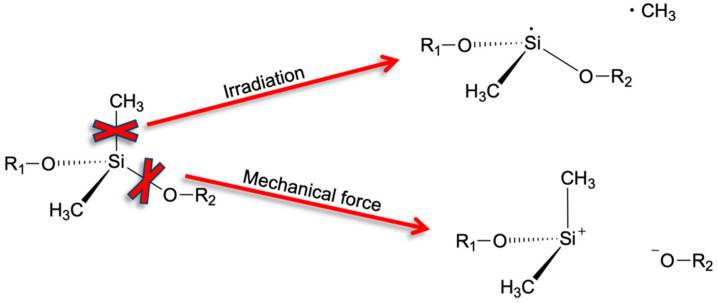
Illustration of silicone degradation via (i) Si-C bond and (ii) Si-O bond cleavage. Both of the illustrated intermediates are rather metastable and subject to (i) radical reactions yielding methane, ethene, etc. (which can be traced by gas sensors) and (ii) addition to adjacent silicone moieties leading to the reorganization of the network of -Si-O-Si- bonds.

Among the different types of silicone oils, polydimethylsiloxane (PDMS) is particularly prominent, both in terms of industrial application and mechanistic characterization. Based on a series of experimental evidence (collected at a broad range of temperature, with/without radiation and oxidative stress), two main mechanisms of PDMS degradation were suggested [6,7,8,14,15]. Mechanism (i) is initiated from homolytic scission of Si-CH_3_ bonds, leading to the formation of radicals that experience cascades of further reaction steps. Among the final products, methane and other hydrocarbons gas formation can be traced as signatures of Si-CH_3_ bond cleavage upon irradiation or high-temperature (~1000 K) pyrolysis. In turn, for the stretching and collisions of silicone molecules, mechanism (ii) must also be considered. It is based on the cleavage of Si-O bonds, thus heterolytic dissociation leading to charged intermediates. Schematics of both mechanistic pictures are illustrated in Figure 1.

In terms of bond dissociation energy of stand-alone molecular moieties, Si-C cleavage (78 kcal/mol) is clearly preferred over Si-O dissociation (108 kcal/mol) [16]. On this basis, we argue that silicone degradation by irradiation mainly follows Si-C bond scission according to mechanism (i). In turn, for damage provoked by pyrolysis or high-pressure it is important to go beyond considerations of bond dissociation in individual molecules. Indeed, collisions of several silicone moieties may lead to simultaneous reaction events that are best described as concerted Si-O bond reorganization rather than cleavage and reformation. To this end, a particularly striking signature of mechanism (ii) is the formation of cyclic PDMS species upon degradation of linear silicone molecules [8,9]. While head-to-tail connection of initially linear silicone molecules would lead to few but rather large rings, PDMS cyclization is typically observed in terms of many, comparably small oligomers. An intuitive explanation for this is given by scission of short strands from loops and concerted re-connection of elsewise dangling -(CH_3_)_2_Si^+^ and -O^−^ moieties into rings [8,9].

To provide in-depth mechanistic characterization, theory based on quantum chemistry and/or molecular mechanics approaches operate at atomic resolution and thus appear particularly promising. While the analysis of (irradiation-induced) scission of bonds in stand-alone molecules is a routine task to quantum chemical calculations, investigations involving the complexity of bulk liquids call for computationally effective modelling approaches. To this end, reactive molecular mechanics models offer insights from direct molecular dynamics simulations of nm-scale fluid models. So far, the ReaxFF method has been employed to suggest degradation products from extreme (2400 K and larger) temperature [17,18,19]. While this mainly refers to fast reactions via the radical-based mechanism (i), in what follows we present a tailor-made reactive molecular mechanics model of PDMS for studying degradation at room temperature. Mimicking extreme pressure changes during oscillatory testing of heavy-duty silicone dampers, we aim at elucidating possible low-temperature routes according to mechanism (ii). Using simulation models of hundreds of silicone oil molecules, we offer unbiased comparison of Si-O bond dissociation, formation and concerted reorganization ranging from scission and fusion of linear PDMS strands to cyclization processes. For this purpose, we take use of a recently presented reactive modelling approach to describing silicone oil formation [20]. To this end, both formation and degradation of silicone oil are treated within analogous simulation setups.

## 2. Methods

Silicone oils can be formulated from condensation reactions of dimethylsilanediol (DMS-D) and trimethylsilanol (TMS), using DMS-D as the base unit of the polymer chains and TMS as terminal groups, respectively. Subject to the relative concentrations of divalent DMS-D and monovalent TMS, the formed PDMS molecules can be tuned in average size but will still feature a broad distribution of polymer length. Thus, it is intuitive that the explicit formulation not only affects the viscosity, but also the ageing of PDMS. Using combined quantum/molecular mechanics (QM/MM)-based molecular dynamics simulations, we recently presented a modelling study of silicone oil formulation from DMS-D/TMS ratios of 100/0, 90/10 and 80/20, respectively [20]. Each of these models comprises a total of 500 DMS-D/TMS which, upon curing (≥99%), reflects different silicone oil formulations in ~4 nm sized simulation cells (featuring 3D periodic boundary conditions). The size of PDMS strands varies from 65 (for DMS-D/TMS = 100/0), 15 (90/10) to 9 (80/20) formulae units, whilst the standard deviations were found as 46, 12 and 9, respectively [20]. In the present work, we adopt these models and compare this to a fluid of 100 cyclopentasiloxane species, namely 5-membered rings of dimethylsilicone (D5)—which is often used as a base oil in cosmetics and hair care applications. Each of these 4 simulation models were quadrupled along the *x*-axis and doubled in the perpendicular directions and subjected to relaxation from 10 ns molecular dynamics simulation at 300 K and 1 atm, to provide extended cells of 16 × 8 × 8 nm dimensions.

The equilibrated, 3D-periodic simulation cells were then subjected to deformation cycles by periodically scaling the box dimensions *L_x_* along the *x*-coordinate:

(1) $$L_{x}\left(t\right)=L_{0}+f\cdot L_{0}\sin\left(\frac{2\pi }{T_{p}}\cdot t\right)$$

where *L*_0_ refers to the equilibrium box size at ambient conditions to which deformation by factor *f* is applied at a period of *T_p_*. In turn, no variation in the box dimensions in the perpendicular directions is allowed. Subject to the choice of *f* and *T_p_*, this allows drastic over- and underpressurization at time scales down to picoseconds—such that we can tailor our cyclic damper setup to enable silicone oil degradation within the ns scale of molecular dynamics simulations. The overall number of formulae units per simulation cell amounts to 8000—which readily provides some assessment of the statistical manifold of possible degradation products. To further confirm the statistical quality of our simulation runs, 3 independent cyclic testing runs were performed for each system (DMS-D/TMS = 100/0, 90/10, 80/20 and D5), respectively. While the D5 systems were simply depicted from different snapshots of the pristine cyclopentasiloxane fluid, for the DMS-D/TMS systems we take use of the 3 independent polymerization runs reported in ref. [20].

The interaction models were fully adopted from our previous studies of silicone formation [21,22]. Likewise, the molecular dynamics simulations were carried out using LAMMPS with a time step of 1 fs and a real-space cutoff of 1.2 nm. Ewald summation is applied to the long-range Coulomb interactions. Constant temperature is imposed via the LAMMPS-based implementation of the Nosé–Hover thermostat using a relaxation time constant of 0.1 ps [23,24]. All data analyses and visualizations were performed using Python 3.12, NetworkX 3.3 and VMD 2.0 [25,26].

## 3. Results

Silicone oils typically reflect a range of molecular compositions even before thermal or mechanical testing is applied. The distribution of linear strands and ring-like moieties, as well as the range of the respective sizes, strongly depends on the specific oil formulation. To assess this issue in an unbiased manner, we depict our simulation models from a previous study which addressed the explicit silicone polymerization process as a function of precursor composition [20]. To one side, this helps to ensure realistic starting points to testing different silicone oil formulations. To the other, we also adopt the underlying interaction models for studying both the dynamics of given silicone molecules and their reactivity. Guided by combined quantum/molecular mechanics approaches our previous work features a ‘reactive’ molecular mechanics model in terms of Si-O bond formation/dissociation [20]. By adopting this interaction model, we consequently investigate the cleavage and reformation/reorganization of Si-O bonds in full analogy to treating the polymerization process.

The ageing of silicone oils (fortunately) is a rather slow process, and the underlying time scales drastically exceed the ns scale inherent to our molecular dynamics simulations. We are bound to rather drastic testing setups as compared to the established experimental procedures—which may induce silicone oil ageing by irradiation or heating to several hundred °C in hundreds to thousands of hours [6,7,8,9,14,15]. In a manner mimicking mechanical testing cycles of solids, we take use of our 3-dimensional periodic simulation cells of silicone oil and impose uniaxial simulation cell deformation whilst maintaining the box dimensions in the perpendicular directions. To this end, 2 adjustable parameters are given by (i) the duration *T_p_* of each box deformation cycle and (ii) the extent *f* of simulation cell elongation/compression (see also Equation (1)). Aiming for performing 1000 testing cycles for each system, we chose *T_p_* = 10 ps and scanned a range of values for *f* to impose oil ageing within feasible computational demands. Upon deformation/elongation of the simulation cells by *f* < 10%, we find no or only marginal reactivity. In turn, a reasonable number of reaction pathways allowing for statistical evaluation was collected for *f* = 10%, respectively.

**Figure 2:**
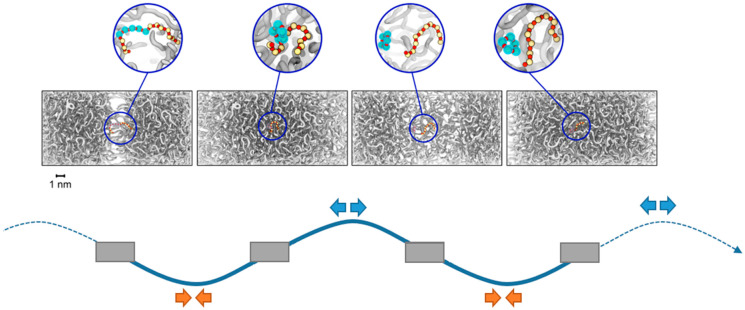
Upper panel: exemplary series of snapshots from mechanical testing of the silicone oil formulation derived from 90/10 DMS-D/TMS. Lower panel: schematics of the alternating compression/elongation cycles applied to the simulation cell. Upon completion of subsequent half-cycles, a linear silicone strand experiences coiling/elongation moves that eventually provoke Si-O bond reorganization in favour of extruding a cyclopentasiloxane species. Note that the overall number of bonds is unchanged and the coordination of Si and O atoms remains balanced. Yet the reaction energy is not exactly zero, but (slightly) endothermic because of the intramolecular strain inhered to the ring formation. For better visibility only Si (yellow/cyan) and O (red) atoms are shown, whilst the remaining oil molecules are illustrated in grey, respectively.

Snapshots from a sequence of testing cycles are depicted in Figure 2. For the sake of comparability, each frame is depicted at *L_x_*(t) = *L*_0_, i.e., the original box dimensions. However, subject to the elongation/deformation imposed before, the half-cycle differs in terms of local density. The illustrated trajectory exemplifies the reorganization of a silicone molecule in the box centre, namely the extraction of a 5-membered ring (D5) from a linear silicone strand.

**Figure 3:**
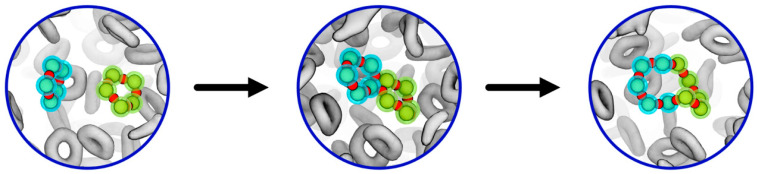
Two cyclopentasiloxane molecules depicted from testing the pure D5 oil formulation. Only the Si (cyan/green) and O (red) atoms are shown for the reacting species, whereas all other molecules are indicated in grey, respectively. While the forward reaction is obviously favoured for the pristine D5 system, the backward reaction is also observed at later stages of cyclic testing once larger amounts of extended rings are available.

The production of cyclic PDMS was observed as a general feature of each of the oil formulations (DMS-D/TMS = 100/0, 90/10 and 80/20) based on mainly linear silicone strands. This nicely reflects the experimentally observed trend towards ring-like molecules upon ageing of silicone oils based on initially linear molecules [6,7,8,9,14,15]. In turn, cyclic PDMS species showed only limited reactivity, namely in terms of fusion/dissociation of small/larger silicone rings (Figure 3). Indeed, even the D5 model system, which initially comprised only 5-membered silicone rings, did not produce any linear moieties upon our testing runs.

**Figure 4:**
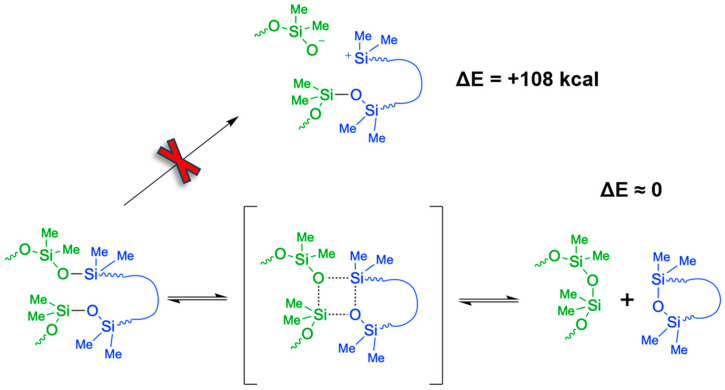
General mechanism for the degradation of PDMS strands. Undercoordinated forms of Si and O atoms are not observed, but the molecules may be temporarily overcoordinated followed by possible bond rearrangements. A cyclic transition state is formed at any position on the strand. It follows a shortening of the chain and the segregation of a cyclic siloxane product. As shown by the simple case of the system with only cyclic siloxane, in which enlargement occurs, this reaction is reversible. This suggests a kinetic driving force rather than a thermodynamical one. This reaction may happen within the same molecule or with other molecules after collision.

An overarching signature of all reactive events is given by the strict avoidance of any under-coordination of bonding partners. We thus argue that neither radicals or dangling -Si(CH_3_)_2_^+^ or -O^−^ moieties are formed. As a predominant mechanism of silicon oil ageing, we instead suggest the concerted association of newly formed Si∙∙O contacts and dissociation of previous Si-O bonds. This encompasses the temporary formation of 4-centred motifs, as illustrated in Figure 4. Upon reaching the product state, this reaction scheme features a fully balanced number of Si-O bonds. Accordingly, the overall change in energy is close to zero—albeit intramolecular strain and intermolecular interactions may provoke some deviation. However, we argue that such contributions are small compared to the rather endothermic +78 and +108 kcal/mol reaction energies related to the mechanisms discussed in Figure 1, respectively.

The overall picture of silicone ageing is best described as a dynamical interplay of addition and extrusion processes. This encompasses ‘linear ↔ linear’ and ‘ring ↔ ring’ pathways, featuring the production of both larger and shorter moieties, such that the broadening of the size distribution of linear/cyclic silicone molecules is observed. If stand-alone, this interplay of chain/ring growth and fragmentation would suggest a dynamic equilibrium. However, the ‘linear → ring’ processes are clearly biased towards ring formation and therefore imply gradual conversion of the linear silicone species to cyclic PDMS.

**Figure 5:**
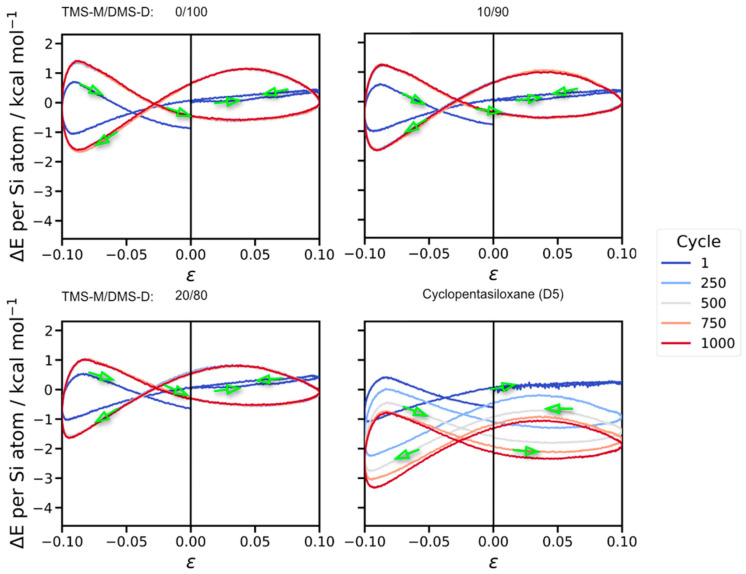
Profiles of potential energy Δ*E*(*t*) = *E*(*t*) − *E*(*t* = 0) as functions of the externally applied deformation *ε* = [*L*(*t*) − *L*_0_]/*L*_0_ of the simulation cells. Each testing cycle is initiated by elongation, followed by compression, etc. (green arrows). While the silicone oil ageing reactions continuously proceed, convergence of the energy profiles is indicative of zero net reaction energy. This is experienced upon a few 100 s of cycles for the linear silicones, whereas the cyclopentasiloxane system gradually converges upon 750–1000 cycles. Mean values taken from three independent runs performed for each setup are shown, and relative errors are within 2–14%, respectively.

To analyze the ageing process in a quantitative manner, it is intuitive to monitor the system energy *E* of our different silicone oil models. This is complicated by the time-dependence of *E* stemming from the oscillatory elongation/compression of the simulation cell. However, the uptake of energy is damped by the thermostat algorithm which drains kinetic energy from the simulation models. Selecting a target temperature of 300 K, we still find the actual system temperature as 300 ± 5 K. Accordingly, it is insufficient to simply consider time averages of the system energy, and we argue that the entire energy profile of the individual testing cycles should be compared. In Figure 5, we demonstrate such comparison for the first testing cycle and cycles 250, 500, 750 and 1000, respectively. While the first testing cycle refers to the pristine oil formulation initially relaxed at 300 K, upon a few tens of testing cycles, our models reach a dynamic equilibrium of heat uptake from the externally induced acceleration and the kinetic energy damping from the thermostat algorithm.

**Figure 6:**
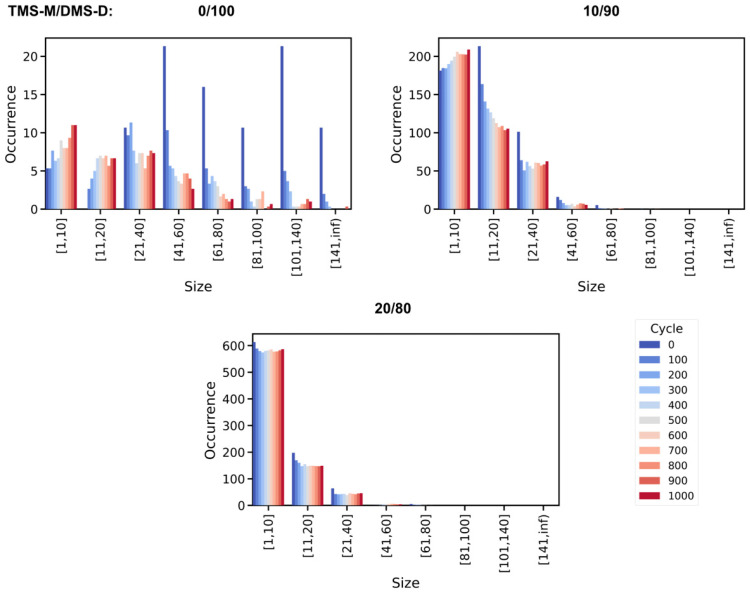
Occurrence distribution of the differently sized (number of Si atoms) *linear* PDMS species as functions of the number of testing cycles experienced. Averages were taken over the three independent runs performed for each of the silicone oil formulations investigated. Note the substantial differences in the size distribution statistics obtained for the different siloxane oil formulations studied.

**Figure 7:**
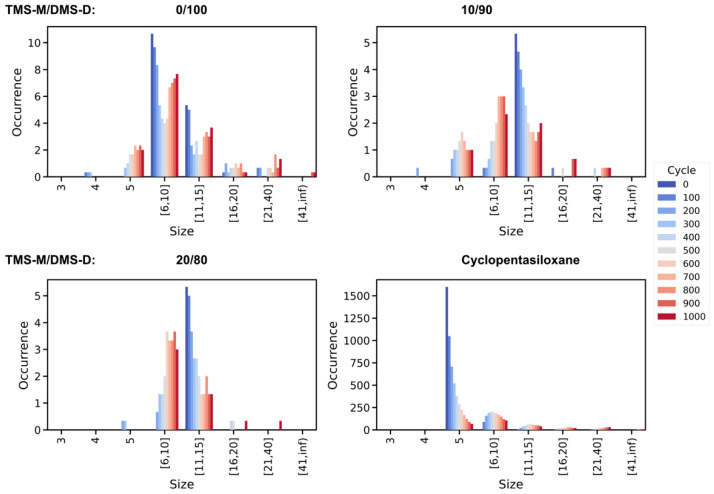
Occurrence distribution of the differently sized (number of Si atoms) *cyclic* PDMS species as functions of the number of testing cycles experienced. Averages were taken over the three independent runs performed for each of the silicone oil formulations investigated. For the linear (TMS-D/DMD-D) silicone oil formulations we find systematic production of cyclic PDMS molecules. In turn, the D5 model evolves to a broad distribution of differently sized rings without producing linear PDMS species.

Indeed, the TMS-M/DMS-D = 0/100, 10/90 and 20/80 models all show consistent energy profiles for cycles 250–1000, respectively, whereas the PDMS molecules experience continuous bond reorganization reactions (Figure 6 and Figure 7). This implies that the underlying silicone oil polymers experience bond reorganization in terms of linear ↔ linear (Figure 6) and linear → ring (at late stages also ring ↔ ring) reactions (Figure 7) without notable change in overall energy. We argue that the conversion of PDMS strands is triggered by momentary strain induced by the testing procedure. Such externally induced strain affects linear and ring-type PDMS moieties in a size-dependent manner—with large strands/rings being particularly prone to bond elongation and eventually dissociation.

While the ageing of linear silicone oil molecules mainly features iso-energetic reactions, we observe a strikingly different behaviour for the D5 model. Indeed, when comparing subsequent testing cycles, we find the cyclopentasiloxane oil system to undergo a continuous reduction in energy. This is reflected by shifting of the corresponding energy profiles which gradually converge upon completion of 1000 testing cycles. We attribute the observed reduction in energy of the pristine D5 silicone oil to the formation of larger rings which are less prone to intramolecular strain. Indeed, when comparing the potential energy of differently sized cyclic PDMS molecules from relaxation in vacuum, we find the average energy per Si atom to continuously decrease with the overall size of the ring. To this end, cyclopentasiloxane is disfavoured by Δ*E* = 3.0 kcal/mol per Si atom with respect to cyclodecasiloxane, whilst an additional 0.7 kcal/mol per Si atom is observed for the 20-membered PDMS species, respectively. In the long run, exhaustive testing is thus expected to favor moderately sized, e.g., 5–25 membered rings (see also Figure 7). Nevertheless, the formation of larger species of cyclic PDMS is energetically assessable. However, in our testing runs, such nm-sized ring species are quite prone to bond dissociation because of the ±10% deformation applied to the simulation cells.

## 4. Conclusions

In summary, the oscillatory testing of silicone oil with molecular dynamics simulations was implemented with a reactive force field to provide insights into the mechanisms of heterolytic bond scission. While we find a manifold (thousands) of reaction events, all of these follow a concerted association of a newly forming Si∙∙O bond and the dissociation of the adjacent Si-O bond within temporary formation of a four-centred motif. Four different silicone oil formulations were explored, with three of these models mimicking mixtures of differently sized linear and (to a minor extend) cyclic PDMS species [20]. On this basis, we clearly identify the formation of cyclic molecules. In turn, as an additional model, we designed pure cyclopentasiloxane oil to confirm the persistence of PDMS rings over linear silicone molecules.

The observed trend to silicone cyclization in the course of ageing is in line with experimental investigations [6,14,15]. While these experiments were based on silicone oil ageing by elevated temperature (600–1000 K) rather than mechanical testing, the formation of small cyclic oligomers ([(CH_3_)_2_SiO]_x_ with x ≤ 12) as main products is reasonably well reproduced by our simulation setup. Interestingly, our simulations performed at 300 K reveal the disfavoring of very small, e.g., 3–4 membered rings, because of intramolecular strain. Upon heating up to T = 1000 K and vaporization of the products, it is, however, quite intuitive to expect increased production of the entropically favoured small-molecule species (since entropy S scales with the logarithm of the number of freely mobile molecules). To this end, the formation of cyclotri- and cyclotetrasiloxanes, respectively, arguably stems from the extreme temperatures applied as the entropy gain may compensate for the less favourable formation energy of these molecules compared to D5.

We argue that our molecular simulation setup offers an additional perspective to understanding the ageing of silicone oil formulations—and thus provide a step forward to rationally designing oil formulations with tailor-made properties. For example, model extension to exploring the role of additives or contaminations is straight-forward. Likewise, different temperature/pressure conditions could be contrasted to provide molecular scale insights into the resulting ageing products.

## Data Availability

The original contributions presented in this study are included in the article. Further inquiries can be directed to the corresponding author.
